# One or Two in a Bed

**Published:** 1892-05

**Authors:** 


### ONE OR TWO IN A BED.

Persons often ask "Is it healthful for two persons to sleep in the same bed?" This same question is varied thus; "Is it healthful for an aged and a very young person to sleep together? If not, which suffers most, the aged or the young person?" We have always answered these questions by saying, No, to the first question. It is always unhealthful for two persons to sleep together in the same bed and under the same covers. The air under the bed-covers immediately surrounding the body of the sleeper is exceedingly impure, becoming more and more impregnated with poisonous substances, escaping through the excretory glands of the skin, from the moment the person retires until he arises. The odor of the bed clothing, after having been occupied for a night, is often positively offensive to the nostrils of a person with an unimpaired sense of smell, especially one who has just come in from out-doors, where the fresh, pure air, has been breathed.

The poisonous character of this under-the-bed-clothes air would be somewhat more likely to affect the susceptible constitution of a child than that of an adult. In elderly persons, the amount of impurities in the air surrounding the sleeper, must be greater than in young persons, consequently, while both persons would be more or less injured, the proportion of harm would doubtless be greater to the young person than to the person of more advanced years. Mr. Treves, of the great London Hospital, has recently called attention to the fact, that wounds, especially of the lower limbs, heal much sooner when kept exposed to the open air, instead of being covered up by bed-clothing. He remarks that the air under the bed-clothing is foul and almost hot, and hence likely to be very harmful to wounds in which it may come in contact. This seems to be a very ample demonstration of the correctness of the views above expressed, to which we have before given expression.

